# Identifying IDH-mutant and 1p/19q noncodeleted astrocytomas from nonenhancing gliomas: Manual recognition followed by artificial intelligence recognition

**DOI:** 10.1093/noajnl/vdae013

**Published:** 2024-02-01

**Authors:** Lei He, Hong Zhang, Tianshi Li, Jianing Yang, Yanpeng Zhou, Jiaxiang Wang, Tuerhong Saidaer, Xiaoyan Bai, Xing Liu, Yinyan Wang, Lei Wang

**Affiliations:** Department of Neurosurgery, Beijing Tiantan Hospital, Capital Medical University, Beijing, People’s Republic of China; Department of Neurosurgery, Beijing Tiantan Hospital, Capital Medical University, Beijing, People’s Republic of China; Department of Neurosurgery, Beijing Tiantan Hospital, Capital Medical University, Beijing, People’s Republic of China; Department of Neurosurgery, Beijing Tiantan Hospital, Capital Medical University, Beijing, People’s Republic of China; Department of Neurosurgery, Beijing Tiantan Hospital, Capital Medical University, Beijing, People’s Republic of China; Department of Neurosurgery, Beijing Tiantan Hospital, Capital Medical University, Beijing, People’s Republic of China; Department of Neurosurgery, Beijing Tiantan Hospital, Capital Medical University, Beijing, People’s Republic of China; Department of Radiology, Beijing Tiantan Hospital, Capital Medical University, Beijing, People’s Republic of China; Department of Pathology, Beijing Tiantan Hospital, Capital Medical University, Beijing, People’s Republic of China; Department of Neurosurgery, Beijing Tiantan Hospital, Capital Medical University, Beijing, People’s Republic of China; Beijing Neurosurgical Institute, Capital Medical University, Beijing, People’s Republic of China; Chinese Institute for Brain Research, Beijing, People’s Republic of China; Department of Neurosurgery, Beijing Tiantan Hospital, Capital Medical University, Beijing, People’s Republic of China; Beijing Neurosurgical Institute, Capital Medical University, Beijing, People’s Republic of China

**Keywords:** astrocytoma, MRI, machine learning, nonenhancing glioma, T2-FLAIR mismatch

## Abstract

**Background:**

The T2-FLAIR mismatch sign (T2FM) has nearly 100% specificity for predicting IDH-mutant and 1p/19q noncodeleted astrocytomas (astrocytomas). However, only 18.2%–56.0% of astrocytomas demonstrate a positive T2FM. Methods must be considered for distinguishing astrocytomas from negative T2FM gliomas. In this study, positive T2FM gliomas were manually distinguished from nonenhancing gliomas, and then a support vector machine (SVM) classification model was used to distinguish astrocytomas from negative T2FM gliomas.

**Methods:**

Nonenhancing gliomas (regardless of pathological type or grade) diagnosed between January 2022 and October 2022 (*N* = 300) and November 2022 and March 2023 (*N* = 196) will comprise the training and validation sets, respectively. Our method for distinguishing astrocytomas from nonenhancing gliomas was examined and validated using the training set and validation set.

**Results:**

The specificity of T2FM for predicting astrocytomas was 100% in both the training and validation sets, while the sensitivity was 42.75% and 67.22%, respectively. Using a classification model of SVM based on radiomics features, among negative T2FM gliomas, the accuracy was above 85% when the prediction score was greater than 0.70 in identifying astrocytomas and above 95% when the prediction score was less than 0.30 in identifying nonastrocytomas.

**Conclusions:**

Manual screening of positive T2FM gliomas, followed by the SVM classification model to differentiate astrocytomas from negative T2FM gliomas, may be a more effective method for identifying astrocytomas in nonenhancing gliomas.

**Key Points**

- T2FM identifies astrocytomas in nonenhancing gliomas with high specificity.
- Artificial intelligence assists in distinguishing astrocytomas from negative T2FM gliomas.
- Manual recognition followed by artificial intelligence recognition is the better choice.

**Importance of the Study**

Previous research has demonstrated that the T2-FLAIR mismatch sign (T2FM) recognizes astrocytomas in specific pathologic subtypes of gliomas (lower-grade gliomas). Our research indicates that the T2FM sign is extremely specific for distinguishing astrocytomas from nonenhancing gliomas, which suggests that the T2FM sign should have broader applicability (nonenhancing gliomas regardless of pathologic type). We demonstrate for the first time that artificial intelligence is a viable method for identifying astrocytomas from negative T2FM sign gliomas. We, therefore, believe that manual recognition of T2FM sign followed by machine learning classification in negative T2FM sign gliomas is better for identifying astrocytomas in nonenhancing gliomas.

Nonenhancing gliomas do not necessarily indicate low-grade gliomas or gliomas with particular molecular subtypes.^1–3^ For these gliomas, surgical resection is the most fundamental treatment, and many neurosurgeons concentrate on achieving the optimal equilibrium between the extent of resection, survival benefits, and protection of brain functions. For gliomas of different molecular subtypes, the extent of resection is significantly different in terms of patient survival benefits.^4–10^ In astrocytoma with isocitrate dehydrogenase (*IDH*) mutations, even small postoperative volumes have a negative impact on patient survival.^5^ Although for oligodendrogliomas with *IDH* mutations and 1p/19q co-deletions, extensive resection does not improve survival and partial residues do not seem to have a significant adverse impact on the patient’s survival, because of the chemosensitive and indolent nature.^4,11^ Consequently, identifying the pathological subtypes of nonenhancing gliomas before surgery may be crucial for surgical planning.

The magnetic resonance imaging (MRI) characteristics of gliomas correlate closely with their molecular information. Recent studies indicated that the T2-FLAIR mismatch sign (T2FM) can predict *IDH*-mutated (*IDHmt*) astrocytoma with nearly 100% specificity and 100% positive predictive value (PPV) among lower-grade gliomas.^12–14^ The T2FM is a visual feature that can be recognized by the naked eye, and it is usually defined by the presence of 2 distinct MRI features: (1) tumor displays the complete or near-complete and almost homogeneous hyperintense signal on T2-weighted images; (2) tumor displays the relatively hypointense signal on the T2-weighted FLAIR sequence except for a hyperintense peripheral rim.^15^ The advantage of T2FM is that it is readily recognized by the observer without complex preprocessing of the original image, and this recognition has good consistency among different observers. However, only 18.18%–56.0% of astrocytomas exhibit a positive T2FM.^12–14,16–18^ Manual identification of astrocytomas from the negative T2FM gliomas is difficult. Radiomic features can quantify massive image representations that are difficult for people to perceive. Using these radiomic features can significantly improve the accuracy of identifying targets using the machine learning method. However, due to its relatively complex operations and the requirement for original digitized images, this method is not always more advantageous than manual recognition. Combining human T2FM recognition with machine learning classification based on radiomic features may allow for the optimal utilization of their merits while mitigating their respective drawbacks.

To better identify astrocytomas from nonenhancing gliomas, this study investigated the following method: the T2FM was first estimated using artificial work, and then machine learning classification was carried out for the negative T2FM gliomas.

## Materials and Methods

**Figure 1.**
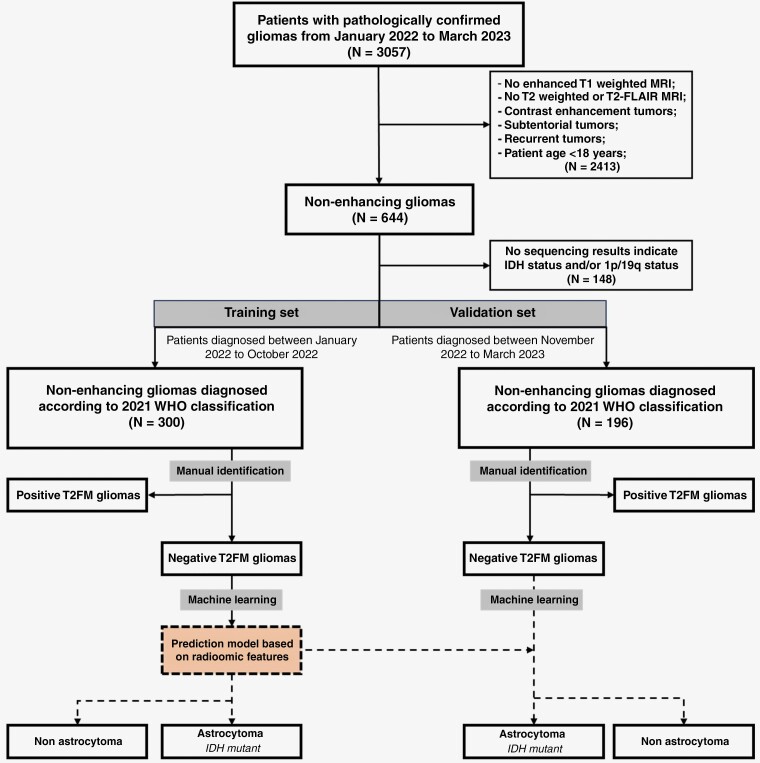
Flowchart of the included patients and research design ideas.

We retrospectively collected all patients with pathologically confirmed gliomas (regardless of pathological type or grade; *N* = 3 057) from January 2022 to March 2023 in Beijing Tiantan Hospital. Patients diagnosed between January 2022 to October 2022 and between November 2022 to March 2023 were assigned to the training set and independent validation set, respectively. The detailed procedure is shown in Figure 1. Conforming to the principles of the Declaration of Helsinki, the institutional review board of Beijing Tiantan Hospital approved this retrospective study.

### MRI Screening

All MR images were obtained on a 3.0 T scanner (GE, Siemens, or Philips). The T2-weighted images were acquired with a repetition time (TR) of 3 100–7 400 ms, echo time (TE) of 86–120 ms, in-plane resolution of 0.3–0.5 mm, and 4.5–5.5 mm slice thickness. FLAIR images were acquired with a TR 4 800–9 000 ms, TE 80–256 ms, inversion time (TI) 1 650–2 500 ms, in-plane resolution of 0.3–0.5 mm, and 4.5–5.5 mm slice thickness. Two observers (a neurosurgeon with 5 years of experience and a radiologist with 8 years of experience) reviewed the MR images independently. Both observers were unaware of the histopathologic and molecular diagnoses of each case. A total of 2 413 cases were eliminated based on the following exclusion criteria: (1) absent enhanced T1-weighted MR image, (2) absent T2-weighted or FLAIR MR image, (3) any degree of enhancement in the tumor region on the enhanced T1-weighted image, (4) tumor location under the tentorium cerebelli, (5) recurrent tumor, (6) receipt of radiotherapy before undergoing an MRI scan, or (7) patient age <18 years old. When 2 observers disagreed, the final determination was made by a neurosurgeon with more than 30 years of clinical experience.

### Pathological Screening

Based on the 2021 WHO classification of tumors of the central nervous system,^19^ a neuropathologist with more than 10 years of experience reevaluated each patient’s pathological results. A total of 148 cases were eliminated due to the lack of sequencing results. Eventually, 496 supratentorial nonenhancing gliomas were included in our research (training set, 300; and validation set, 196).

### T2FM Assessment in the Training Set

The T2FM evaluation criteria were derived from previous research and categorized as (1) classic T2FM: complete or near-complete and homogeneous hyperintense signal on T2-weighted imaging, hypointense signal in almost whole tumor region on FLAIR except for a hyperintense peripheral rim; (2) geographic T2FM: heterogeneous appearance on T2-weighted signal with a mirroring hypointense FLAIR pattern and a hyperintense distinct peripheral rim component on FLAIR image; (3) negative T2FM: not meeting the above conditions.^12,15,20^ Two trained observers (a neurosurgeon with 5 years of experience and a radiologist with 8 years of experience) who were unaware of the histopathologic and molecular diagnoses independently evaluated each of the 300 patients in the training set for the T2FM according to the assessment criteria. The following rules were applied when2 observers evaluated the same case differently: (1) if 1 observer’s evaluation result was the classic T2FM and the other’s was the geographic T2FM, the conclusion was the geographic T2FM; and (2) if 1 observer’s evaluation result was the classic T2FM or geographic T2FM and the other’s was the negative T2FM, the conclusion was the negative T2FM.

Statistical analysis was performed using MATLAB. The Kruskal–Wallis H test was used for the patient’s age. The Chi-square test or Fisher’s exact test was applied for ordinal and categorical variables. Interobserver agreement was assessed using Cohen’s kappa statistic (κ); κ values ≤ 0.2 indicated slight agreement, 0.21–0.4 indicated general agreement, 0.41–0.6 indicated moderate agreement, and >0.6 indicated basic agreement.^21^ The *P* value <.05 was considered statistically significant.

### Extraction and Selection of Radiomics Features From Negative T2FM Sign Gliomas in the Training Set

**Figure 2.**
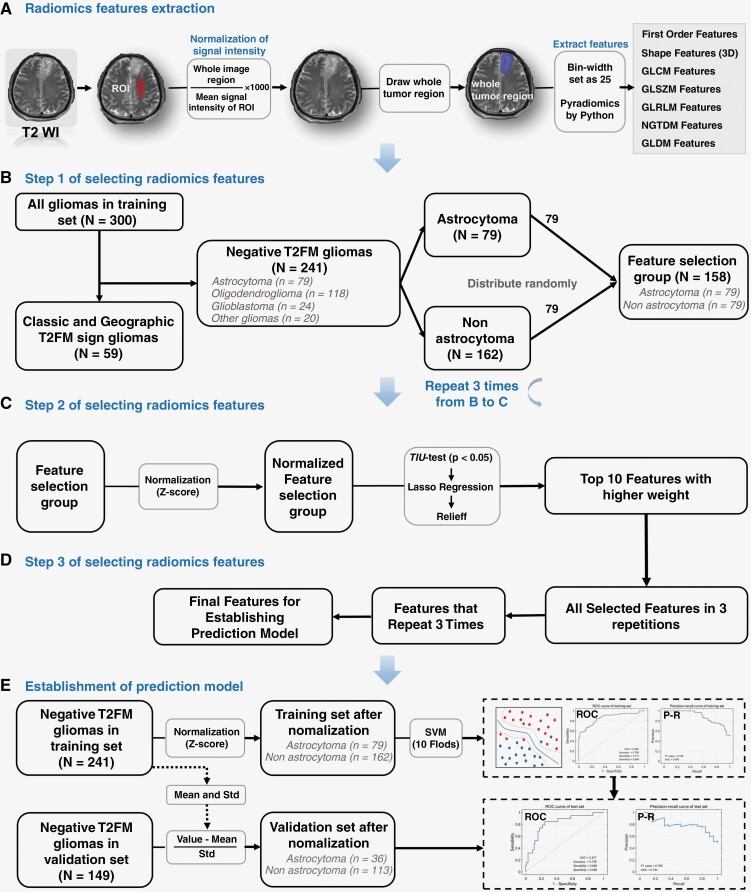
Step diagram for establishing multiparameter prediction model. (**A**) MR images preprocessing and radiomics features extraction, (**B**–**D**) features selection, and (**E**) establishment and validation of predictive models. SVM, Support vector machine; ROC, receiver-operating characteristic curve; P-R, precision recall curve; SD, standard deviation.

The T2-weighted images were first subjected to N4 bias correction. To reduce the variance in signal intensity values between T2-weighted images, standardization was performed for images (entire image region divided by the mean signal intensity of the reference ROI, then multiplied by 1 000). The reference ROI was marked in the normal semiovale center. Then, all images were resized to 256 × 256, and the size of the voxels was resampled to 1 × 1 × 1 mm. The mask of the entire tumor was manually segmented by a neurosurgeon and a radiologist, independently, and confirmed by a senior neurosurgeon (Figure 2A). This preprocessing was performed in MATLAB (R2021a, The Mathworks, Inc., Natick, MA, USA) using the Multimodal Radiomics Platform (http://cflu.lab.nycu.edu.tw/MRP_MLinglioma.html). The radiomic features of the entire tumor region were extracted using the “Pyradiomics” package by Python.^22^

A total of 241 cases (or 80.34%) in the training set were classified as negative T2FM gliomas; of these, 162 were nonastrocytomas and 79 were astrocytomas. To prevent errors due to data mismatch, a feature selection process was conducted using 79 astrocytomas and 79 cases selected at random from 162 nonastrocytomas (Figure 2B). The radiomics features of the feature selection group were standardized using the *z*-score transformation. For feature selection, the T/Mann–Whitney U test, LASSO regression, and Relieff were used. The 10 features with the higher weight were collected (Figure 2C). Three repetitions from procedure B (Figure 2B) to procedure C (Figure 2C) were conducted. To ensure the stability of the representativeness of the selected features, we collected the radiomic features that appeared in all 3 repetitions to establish the prediction model (Figure 2D).

### Establishing a Prediction Model in the Training Set

The prediction model was established using selected radiomic features only or radiomic features with clinical information (age and/or gender) of patients. Radiomic features of the training set were normalized via *z*-score transformation. A support vector machine was used to establish the prediction model (a radial basis kernel was used). The 10-fold cross-validation was used to reduce overfitting. The area under the curve (AUC) of the receiver-operating characteristic curve (ROC) and precision recall curve (P-R curve), accuracy, sensitivity, and specificity were used to evaluate the performance of the prediction model (Figure 2E).

### Evaluation With Validation Set

Two trained observers (a neurosurgeon with 5 years of experience and a radiologist with 8 years of experience) who were unaware of the histopathologic and molecular diagnoses independently evaluated each of the 196 patients in the validation set for the T2FM according to the assessment criteria above and categorized it as classic T2FM, geographic T2FM, or negative T2FM glioma.

For negative T2FM glioma in the validation set, the same as the processing of the training set, N4 correction, signal intensity standardization, voxel size resampling, and segmentation of the entire tumor mask were performed, and then the radiomic features of the tumor area were extracted in T2-weighted imaging. z-Score normalization was subsequently applied to the extracted radiomic features in the validation set, utilizing the mean value and standard deviation of the corresponding features from the training set as a basis. The validation set is classified utilizing the prediction model constructed from the training set. The AUC of the ROC and P-R curve, accuracy, sensitivity, and specificity were used to evaluate the performance of the prediction model in the validation set (Figure 2E).

### Refine the Effectiveness of Prediction Models

**Figure 3.**
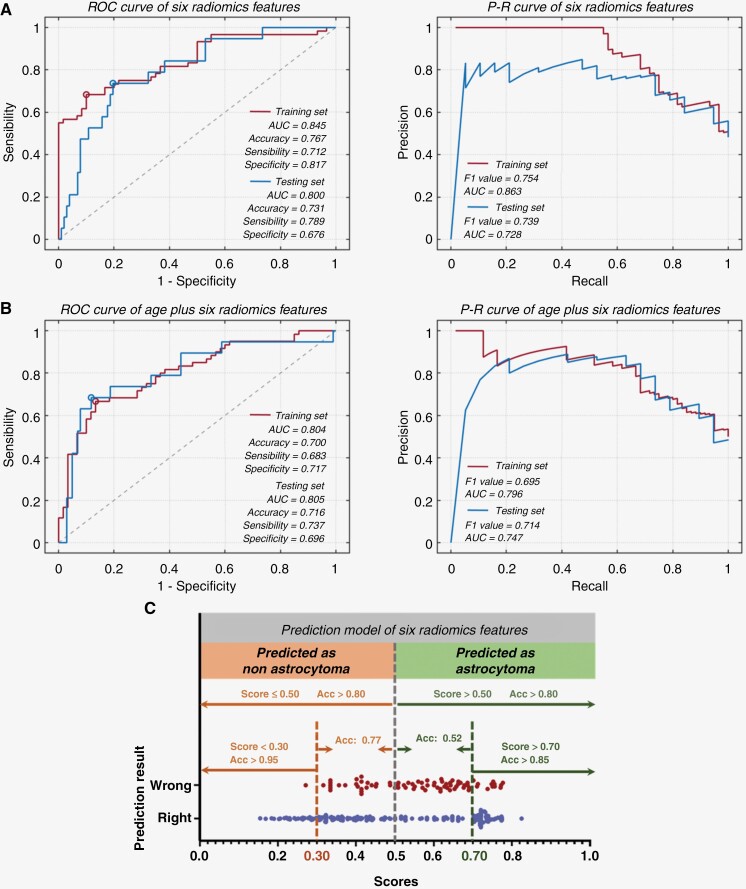
Performance analysis of predictive model. (**A**) Receiver-operating characteristic (ROC) curves and precision recall (P-R) curve for prediction of astrocytomas by radiomic features. (**B**) Receiver-operating characteristic (ROC) curves and precision recall (P-R) curve for prediction of astrocytomas by radiomic features plus patient’s age. (**C**) Model-based predictive scoring for model credibility evaluation. Acc: accuracy.

We performed a segmentation analysis based on the estimated score values that the prediction model estimated to determine the circumstances under which it has better confidence. We first divided the prediction scores derived by the prediction model for the instances in the training and validation sets into score bands, and then evaluated the performance of the prediction model in each of these score bands (Figure 3C).

## Results

### T2-FLAIR Mismatch Sign in Predicting Astrocytoma in Nonenhancing Gliomas With High Specificity But With Low Sensitivity

In the training set, the interobserver agreement measurements for the T2FM had a κ value of 0.858 (Supplementary Table 1). The classic T2FM, geographic T2FM, and negative T2FM were observed in 37 patients (12.33%), 22 patients (7.33%), and 241 patients (80.34%), respectively. The median ages of the patients with classic T2FM, geographic T2FM, and negative T2FM were 39 (22–60), 38 (20–54), and 43 (18–74) years, respectively, and no significant difference was observed (*P* = .099). The difference in the distribution of T2FM between males and females was not statistically significant (*P* = .124). There was a significant difference in the distribution of T2FM between astrocytomas and oligodendrogliomas (*P* < .001). Moreover, there were significant differences in the distribution of T2FM between astrocytomas and glioblastomas (*P* < .001) and between astrocytomas and gliomas of other pathological types (*P* < .001). The 37 cases that presented the classic T2FM and the 22 cases that presented the geographic T2FM were confirmed as astrocytomas. Details are shown in Table 1. In predicting astrocytomas, the sensitivity, specificity, PPV, and negative predictive value (NPV) were 26.81%, 100.00%, 100.00%, and 61.60%, respectively, for the classic T2FM and 42.75%, 100.00%, 100.00%, and 67.22%, respectively, when combining the classic T2FM with the geographic T2FM. Details are shown in Table 2. In the validation set, results were similar to the training set (Tables 1 and 2).

**Table 1.** The Information of Patients in Training Set and Validation Set

	Cohort, No. (%)				
Features	All	Classic T2FM	Geographic T2FM	Negative T2FM	*P* value
Training set					
Patient	300	37 (12.33)	22 (7.33)	241 (80.34)	—
Median age (range)	42 (18–74)	39 (22–60)	38 (20–54)	43 (18–74)	.099^*^
Gender					.124^†^
Men	159	17 (10.69)	16 (10.06)	126 (79.25)	—
Women	141	20 (14.18)	6 (4.26)	115 (81.56)	—
Pathology					<.001^‡^
Astrocytoma	138	37 (26.81)	22 (15.94)	79 (57.25)	—
Oligodendroglioma	118	0 (0.00)	0 (0.00)	118 (100.00)	<.001^§^
Glioblastoma	24	0 (0.00)	0 (0.00)	24 (100.00)	<.001^||^
Other gliomas	20	0 (0.00)	0 (0.00)	20 (100.00)	<.001^¶^
Validation set					
Patient	196	39 (19.90)	8 (4.08)	149 (79.02)	—
Median age (range)	42 (19–69)	37 (19–69)	43 (31–48)	43 (19–69)	.132*
Gender					.020^†^
Men	121	17 (14.05)	4 (3.31)	100 (82.64)	—
Women	75	22 (29.33)	4 (5.33)	49 (65.34)	—
Pathology					<.001^‡^
Astrocytoma	83	39 (46.99)	8 (9.64)	36 (43.37)	—
Oligodendroglioma	89	0 (0.00)	0 (0.00)	89 (100.00)	<.001^§^
Glioblastoma	18	0 (0.00)	0 (0.00)	18 (100.00)	<.001^||^
Other gliomas	6	0 (0.00)	0 (0.00)	6 (100.00)	<.001^¶^

*Note*: *Kruskal–Wallis H test.

^†^Chi-square test.

^‡^Fisher’s exact test.

^§^Astrocytoma versus Oligodendroglioma.

^||^Astrocytoma versus Glioblastoma.

^¶^Astrocytoma versus Other gliomas; Details of other gliomas were seen in Supplementary Table 2.

**Table 2.** The Diagnostic Efficacy of T2FM Sign for IDH-mutant Astrocytoma in Training Set and Validation Set

T2FM sign	IDH-mutant astrocytoma		Sensibility (%)	Specificity (%)	PPV (%)	NPV (%)
	Yes	No				
Training set						
Classic			26.81	100.00	100.00	61.60
Yes	37	0				
No	101	162				
Classic and geographic			42.75	100.00	100.00	67.22
Yes	59	0				
No	79	162				
Validation set						
Classic			46.99	100.00	100.00	71.94
Yes	39	0				
No	44	113				
Classic and geographic			56.62	100.00	100.00	75.84
Yes	47	0				
No	36	113				

*Note*: NPV = negative predictive value; PPV = positive predictive value.

### Machine Learning Classification Could Help in Distinguishing Astrocytomas and Nonastrocytomas in Nonenhancing Gliomas With Negative T2FM

For each case, 1 427 radiomic features were extracted in the T2-weighted image in total. Throughout the 3 screening processes, 6 radiomic features were repeated 3 times and were used for the final model establishment (Supplementary Table 3). For patients with negative T2FM in the training set, the age of patients with astrocytomas differs significantly from that of patients with nonastrocytomas (*P* < .01). However, no significant difference in gender was observed (*P* = .72; Table 3). For the prediction model established using radiomic features only, the AUC, accuracy, sensitivity, and specificity for predicting astrocytomas among negative T2FM gliomas in the training and validation sets were 0.845/0.800, 0.767/0.731, 0.712/0.789, and 0.817/0.676, respectively. The F1 value and AUC of the P-R curve in the training and validation set were 0.754/0.739, and 0.863/0.728, respectively (Figure 3A). For the prediction model established using radiomics features plus patient’s age, the AUC, accuracy, sensitivity, and specificity for predicting astrocytomas among negative T2FM gliomas in the training and validation sets were 0.804/0.805, 0.700/0.716, 0.683/0.737, and 0.717/0.696, respectively. The F1 value and AUC of the P-R curve in the training and validation set were 0.0.695/0.796, and 0.714/0.747, respectively (Figure 3B). We ultimately chose to implement the model that was developed only with radiomic features after a comprehensive assessment of the performance of both models. In identifying astrocytomas, the overall accuracy (score > 0.50) of the prediction model was over 70%. When the score is between 0.5 and 0.7 (26.30% of cases), the accuracy of identifying astrocytomas is only 52%, while when the score is above 0.7 (31.23% of cases), the accuracy exceeds 85%. When the score is between 0.3 to 0.5 (33.01% of cases) and below 0.3 (9.46% of cases), the accuracy of the model in identifying nonastrocytomas is 77% and above 95%, respectively (Figure 3C).

**Table 3.** The Information of Patients with negative T2FM in Training Set

	Astrocytoma	Nonastrocytoma	*P* value
Age (median, range)	37 (18–59)	46 (18–74)	<.01*
Gender (men, %)	40 (50.63%)	86 (53.09%)	.72^†^

*Note*: *Mann–Whitney *U* test.

^†^Chi-square test.

## Discussion

IDH-mutant and 1p/19q codeleted information plays a significant role in assessing the growth rate of gliomas and the prognosis of patients.^23–26^ In contrast to oligodendrogliomas and glioblastomas, expanded resection of astrocytomas may provide greater survival advantages for patients.^7–10^ Therefore, knowing the tumor is an astrocytoma before surgery may help neurosurgeons make a more reasonable surgical plan. Previous studies indicated that the T2FM predicts astrocytomas with practically perfect specificity (100%) and PPV (100%) in adult patients.^13,14,16–18,27–30^ However, 2 considerations cannot be overlooked: first, the vast majority of prior research was restricted to a few pathological subtypes and grades of glioma; second, according to certain studies, the T2FM is also observed in oligodendrogliomas and other types of glioma, and these types of gliomas resemble astrocytomas on MRI.^31,32^ This might lead to an erroneous estimate of the accuracy of the T2FM for predicting astrocytomas in the “real world.” Initially, T2FM was defined as (1) a complete or near-complete and homogeneous hyperintense signal in a T2-weighted image; and (2) a hypointense signal except for a hyperintense thin peripheral rim in FLAIR.^12,15^ However, the research of Throckmorton et al. indicated that the homogeneity in T2-weighted images not be an essential requirement.^20^ Based on the homogeneity in T2-weighted images, they classified T2FM as either classic T2FM or geographic T2FM in their research and observed that both types of T2FM had a 100% accuracy in predicting *IDHmt* astrocytoma. In our analysis, we continually included 496 adult supratentorial nonenhancing gliomas (any pathological type and grade; training set, 300; validation set, 196) from a single center. All 76 cases (training set, 37; validation set, 39) with the classic T2FM were pathologically confirmed to be astrocytomas. After considering different pathological types of glioma, the specificity and the PPV of the classic T2FM sign for predicting astrocytomas remained at 100% in our research. Even though we did not find any false-positive cases in our cohort, previous research has shown that oligodendroglioma and embryonic dysplastic neuroepithelial tumors may exhibit the classic T2FM.^31,32^ However, due to the rarity of oligodendroglioma with classic T2FM and embryonic dysplastic neuroepithelial tumor in adult patients, we believe that the classic T2FM continues to have high predictive accuracy for astrocytomas in the “real world.” All 30 tumors (training set, 22; validation set 8) presenting with a geographic T2FM were pathologically confirmed to be astrocytomas, demonstrating that the geographic T2FM also appears to be a viable predictor of astrocytomas. The sensitivity (10.9%–51%) and NPV (3%–76%) of the T2FM for predicting astrocytomas were poor in previous studies.^12,13,16,29^ Similarly, in our training set and validation set, the sensitivity and NPV for the classic T2FM were 26.81%/46.99% and 61.60%/71.94%, respectively. When the classic T2FM and geographic T2FM were merged, the sensitivity and NPV were 42.75%/56.62% and 67.22%/75.84%, respectively. How to further identify astrocytomas from these negative T2FM gliomas is the key to improving sensitivity and NPV.

Previous studies have shown that astrocytomas and oligodendrogliomas exhibit heterogeneous radiomic features, and machine learning can significantly improve the identification of the 2 tumors.^33–35^ In our research, we used 6 radiomic features to establish a prediction model, we found that the established model with the AUC, accuracy, sensitivity, and specificity were 0.845/0.800, 0.767/0.731, 0.712/0.789, and 0.817/0.676 in the training set and validation set, respectively. Each of these 6 features represents a texture feature of the image, this is consistent with the phenomenon that oligodendrogliomas and astrocytomas exhibit different signal uniformity on T2WI. However, at present, it is unknown precisely why astrocytomas and oligodendrogliomas exhibit significant differences in signal uniformity on T2WI. Our model performs inadequately compared to the results of many previous studies. There could be 2 significant causes for this. First, our research subjects excluded positive T2FM cases, and this screening may have significantly decreased the overall disparity between astrocytoma and nonastrocytoma cases; Second, unlike previous research, we included not only astrocytoma and oligodendroglioma, but also glioblastoma and numerous other glioma types. This more realistic method of inclusion may dilute the overall difference. To know under what conditions the prediction model has higher credibility, we conducted a segmented analysis based on the predicted score values returned by the model. It was ultimately found that when the prediction score was above 0.7 or below 0.3, the accuracy of the model in predicting astrocytoma or nonastrocytoma significantly improved compared to the overall level, with accuracy greater than 0.85 and 0.95, respectively. To ascertain the prediction model’s generalization ability, we conducted additional experiments using image data collected from the cancer imaging archive (TCIA, a public database, the ID of the included image data is shown in Supplementary Table 4). The ultimate outcomes demonstrated that the model’s generalization ability was significantly inadequate (Supplementary Figure 1). And the situation did not improve despite multiple attempts at re-modeling. We think that there are numerous factors contributing to this situation. One of the most important variables contributing to this phenomenon is the considerable variation in MRI scanning parameter settings across different medical centers, such as the selection of imaging technology, the setting of ET, repetition time, and layer thickness of scanning. Variations in these parameters have resulted in distinct abiotic factors’ effects, and at present, there appears to be no effective strategy to eliminate these abiotic factors’ effects completely.^36^ The solution to this problem requires the development of better image processing techniques or the direct use of quantitative imaging techniques such as T2 relaxation time imaging. Second, completely avoiding deviations during the process of manually selecting features is also challenging. Based on our findings, it is feasible to use machine learning to distinguish astrocytomas from negative T2FM gliomas. However, given that it is still difficult to generate accurate predictions for approximately 60% of negative T2FM gliomas in our own data set and the poor generalization ability, clinical implementation of our predictive model remains distant. Additional investigation is necessary to optimize predictive models or consider the potential implementation of deep learning methods to improve the identification of astrocytomas from negative T2FM gliomas.

**Figure 4.**
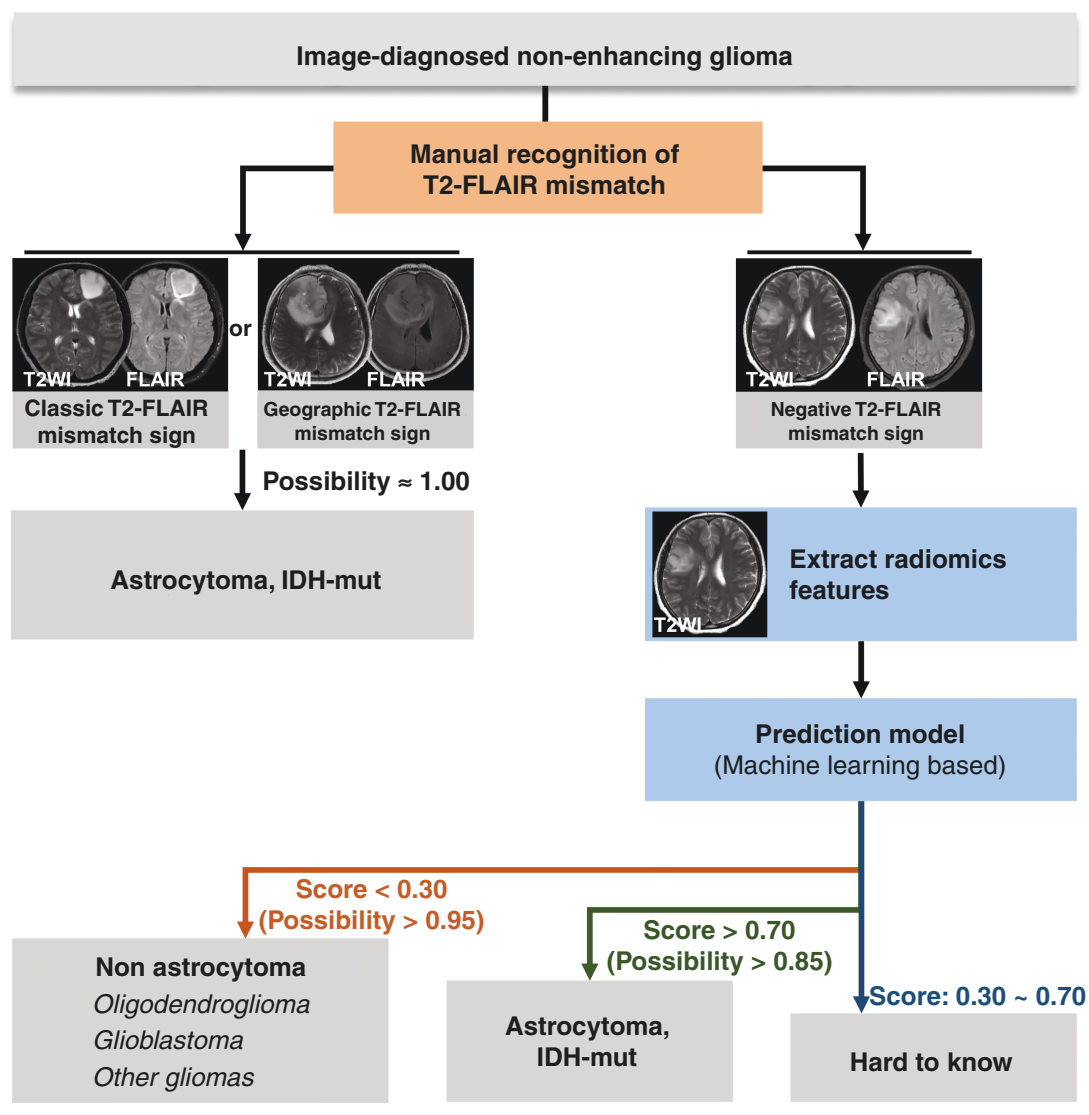
Diagnostic flowchart for identifying astrocytoma from nonenhancing gliomas.

Currently, almost all research (including our research) indicated that the T2FM in predicting astrocytoma with nearly 100% specificity and PPV. More importantly, the T2FM could be easily and quickly recognized by observers based on visual impression and has considerable interobserver consistency. However, it cannot be ignored that not all astrocytomas exhibit T2FM. According to our knowledge, this is the first report to attempt to identify astrocytoma from negative T2FM gliomas. Radiomic features and machine learning seem to be a promising approach to distinguish astrocytomas from negative T2FM gliomas more accurately, however, this requires a relatively complex operational process. Based on our research, we propose a more scientific diagnostic procedure involving the manual screening of nonenhancing gliomas with positive T2FM (classic T2FM and geographic T2FM), followed by the application of machine learning techniques to distinguish astrocytomas from gliomas with negative T2FM (Figure 4).

On the basis of our own central data, this study’s prediction model demonstrated some utility, but overall, the performance of our prediction model is comparatively weak, and the model’s prognostic ability must be improved in the future. In future research, developing a treatment and diagnosis model that incorporates artificial intelligence with clinicians should be a greater focus, as opposed to endeavoring to have artificial intelligence address every problem. To provide an example, ring-like enhancement is identified in around 50% of gliomas diagnosed in adult patients, with an almost perfect prediction of high-grade gliomas (glioblastomas). Moreover, this ring-like enhancement is readily and expeditiously detectable by clinicians (within seconds). It appears illogical for artificial intelligence to incorporate this fraction of ring-enhanced tumors into classification studies. The recognition of an image by artificial intelligence requires the completion of several preprocessing steps, which is significantly more complex and requires a longer amount of time in comparison to the recognition process carried out by human vision. The aim of our study is to construct a diagnostic model for glioma subtype identification that is both pragmatic and logically sound through the integration of manual T2FM recognition and machine learning classification. Undoubtedly, our current diagnostic model is not well developed; however, we maintain our optimism that further investigation in this field will enable us to more effectively exploit the benefits of both human and artificial intelligence.

## Conclusions

Positive T2FM (classic T2FM and geographic T2FM) accurately identifies astrocytomas from nonenhancing gliomas with 100% specificity and PPV. A machine learning approach based on the radiomics features may further help identify astrocytomas from negative T2FM gliomas.

## Supplementary Material

vdae013_suppl_Supplementary_Table_S1

vdae013_suppl_Supplementary_Table_S3

vdae013_suppl_Supplementary_Table_S4

vdae013_suppl_Supplementary_Figure_S1

vdae013_suppl_Supplementary_Table_S2
