# Supplementary material for: Identifying IDH-mutant and 1p/19q noncodeleted astrocytomas from nonenhancing gliomas: Manual recognition followed by artificial intelligence recognition
Source: Neurooncol Adv. 2024 Feb 1;6(1):vdae013. doi: 10.1093/noajnl/vdae013 (PMC10894653; doi:10.1093/noajnl/vdae013)
Supplement: vdae013_suppl_Supplementary_Table_S1 [file vdae013_suppl_supplementary_table_s1.docx]

**Supplementary Table S1**. The results of interobserver consistency analysis in training set.

|  |  | Observer 1 | | |  |  |  |
| --- | --- | --- | --- | --- | --- | --- | --- |
|  |  | Classic | Geographic | Negative | All | *κ value* | *p value* |
| Observer 2 | Classic | 37 | 2 | 0 | 39 | 0.852 | < 0.001 |
|  | Geographic | 3 | 17 | 5 | 25 |  |  |
|  | Negative | 0 | 6 | 230 | 236 |  |  |
|  | All | 40 | 25 | 235 | 300 |  |  |
