# Supplementary material for: Identifying IDH-mutant and 1p/19q noncodeleted astrocytomas from nonenhancing gliomas: Manual recognition followed by artificial intelligence recognition
Source: Neurooncol Adv. 2024 Feb 1;6(1):vdae013. doi: 10.1093/noajnl/vdae013 (PMC10894653; doi:10.1093/noajnl/vdae013)
Supplement: vdae013_suppl_Supplementary_Table_S3 [file vdae013_suppl_supplementary_table_s3.docx]

**Supplementary Table S3**. The features that recurred throughout the three feature selection processes.

| **NO.** | **Image processing** | **Feature name** | **First screening** | **Second screening** | **Third screening** | **Used for modeling** |
| --- | --- | --- | --- | --- | --- | --- |
| 1 | Exponential | GLRLM_Long Run High Gray Level Emphasis | + | + | + | Yes |
| 2 | Log-sigma-1-0-mm-3D | GLCM_Informational Measure of Correlation (IMC) 1 | + | + | + | Yes |
| 3 | Log-sigma-1-0-mm-3D | GLCM_Informational Measure of Correlation (IMC) 2 | + | + | + | Yes |
| 4 | Log-sigma-1-0-mm-3D | GLDM_Dependence Variance | + | + | + | Yes |
| 5 | Original | GLCM_Informational Measure of Correlation (IMC) 1 | + | + | + | Yes |
| 6 | Square | GLDM_Large Dependence High Gray Level Emphasis | + | + | + | Yes |
| 7 | Square | GLRLM _Run Entropy | + |  |  | No |
| 8 | Squareroot | GLDM _Large Dependence High Gray Level Emphasis | + |  |  | No |
| 9 | Wavelet-HLH | GLDM _Dependence Entropy | + |  | + | No |
| 10 | Wavelet-HLH | GLSZM_Small Area Emphasis | + | + |  | No |
| 11 | Log-sigma-1-0-mm-3D | Firstorder_Skewness |  | + |  | No |
| 12 | Squareroot | GLRLM _Short Run Low Gray Level Emphasis |  | + | + | No |
| 13 | Wavelet-LHL | GLDM _Dependence Variance |  | + |  | No |
| 14 | Squareroot | GLDM _Large Dependence High Gray Level Emphasis |  |  | + | No |
| 15 | Squareroot | Firstorder_Variance |  |  | + | No |

GLRLM: Gray Level Run Length Matrix; GLCM: Gray Level Co-occurrence Matrix; GLDM: Gray Level Dependence Matrix; GLSZM: Gray Level Size Zone Matrix.
