# Supplementary material for: Identifying IDH-mutant and 1p/19q noncodeleted astrocytomas from nonenhancing gliomas: Manual recognition followed by artificial intelligence recognition
Source: Neurooncol Adv. 2024 Feb 1;6(1):vdae013. doi: 10.1093/noajnl/vdae013 (PMC10894653; doi:10.1093/noajnl/vdae013)
Supplement: vdae013_suppl_Supplementary_Table_S4 [file vdae013_suppl_supplementary_table_s4.docx]

**Supplementary Table S5**. The ID number of non-enhancing adult-type diffuse gliomas in TCIA.

| Order number | ID number | Order number | ID number |
| --- | --- | --- | --- |
| 01 | UCSF-PDGM-0231 | 36 | UCSF-PDGM-0298 |
| 02 | UCSF-PDGM-0232 | 37 | UCSF-PDGM-0302 |
| 03 | UCSF-PDGM-0233 | 38 | UCSF-PDGM-0326 |
| 04 | UCSF-PDGM-0234 | 39 | UCSF-PDGM-0327 |
| 05 | UCSF-PDGM-0235 | 40 | UCSF-PDGM-0349 |
| 06 | UCSF-PDGM-0236 | 41 | UCSF-PDGM-0351 |
| 07 | UCSF-PDGM-0237 | 42 | UCSF-PDGM-0352 |
| 08 | UCSF-PDGM-0238 | 43 | UCSF-PDGM-0436 |
| 09 | UCSF-PDGM-0240 | 44 | UCSF-PDGM-0438 |
| 10 | UCSF-PDGM-0242 | 45 | UCSF-PDGM-0439 |
| 11 | UCSF-PDGM-0244 | 46 | UCSF-PDGM-0440 |
| 12 | UCSF-PDGM-0245 | 47 | UCSF-PDGM-0441 |
| 13 | UCSF-PDGM-0246 | 48 | UCSF-PDGM-0442 |
| 14 | UCSF-PDGM-0247 | 49 | UCSF-PDGM-0443 |
| 15 | UCSF-PDGM-0248 | 50 | UCSF-PDGM-0444 |
| 16 | UCSF-PDGM-0249 | 51 | UCSF-PDGM-0445 |
| 17 | UCSF-PDGM-0250 | 52 | UCSF-PDGM-0446 |
| 18 | UCSF-PDGM-0251 | 53 | UCSF-PDGM-0448 |
| 19 | UCSF-PDGM-0252 | 54 | UCSF-PDGM-0449 |
| 20 | UCSF-PDGM-0254 | 55 | UCSF-PDGM-0456 |
| 21 | UCSF-PDGM-0255 | 56 | UCSF-PDGM-0465 |
| 22 | UCSF-PDGM-0256 | 57 | UCSF-PDGM-0474 |
| 23 | UCSF-PDGM-0257 | 58 | UCSF-PDGM-0475 |
| 24 | UCSF-PDGM-0258 | 59 | UCSF-PDGM-0476 |
| 25 | UCSF-PDGM-0259 | 60 | UCSF-PDGM-0477 |
| 26 | UCSF-PDGM-0261 | 61 | UCSF-PDGM-0478 |
| 27 | UCSF-PDGM-0262 | 62 | UCSF-PDGM-0482 |
| 28 | UCSF-PDGM-0264 | 63 | UCSF-PDGM-0483 |
| 29 | UCSF-PDGM-0265 | 64 | UCSF-PDGM-0485 |
| 30 | UCSF-PDGM-0266 | 65 | UCSF-PDGM-0490 |
| 31 | UCSF-PDGM-0267 | 66 | UCSF-PDGM-0500 |
| 32 | UCSF-PDGM-0268 | 67 | UCSF-PDGM-0501 |
| 33 | UCSF-PDGM-0272 | 68 | UCSF-PDGM-0534 |
| 34 | UCSF-PDGM-0274 | 69 | UCSF-PDGM-0540 |
| 35 | UCSF-PDGM-0277 |  |  |
