# Supplementary figures and images for: Identifying IDH-mutant and 1p/19q noncodeleted astrocytomas from nonenhancing gliomas: Manual recognition followed by artificial intelligence recognition
Source: Neurooncol Adv. 2024 Feb 1;6(1):vdae013. doi: 10.1093/noajnl/vdae013 (PMC10894653; doi:10.1093/noajnl/vdae013)

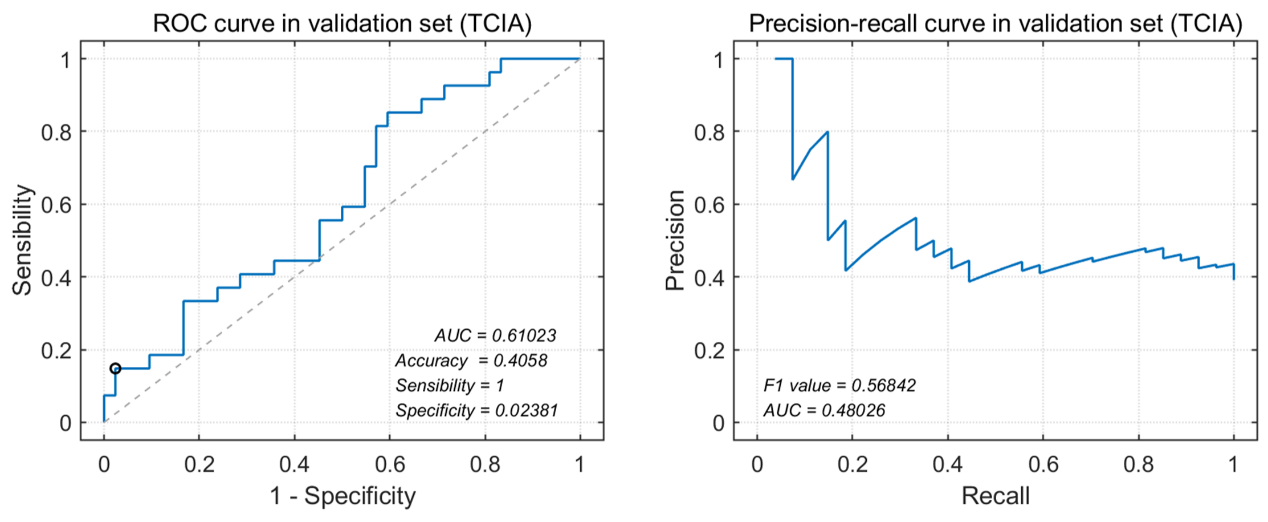


Supplementary figure1: The prediction results of TCIA data by imagomics prediction model.

Supplement: vdae013_suppl_Supplementary_Figure_S1 [file vdae013_suppl_supplementary_figure_s1.docx]
