# Supplementary material for: Identifying IDH-mutant and 1p/19q noncodeleted astrocytomas from nonenhancing gliomas: Manual recognition followed by artificial intelligence recognition
Source: Neurooncol Adv. 2024 Feb 1;6(1):vdae013. doi: 10.1093/noajnl/vdae013 (PMC10894653; doi:10.1093/noajnl/vdae013)
Supplement: vdae013_suppl_Supplementary_Table_S2 [file vdae013_suppl_supplementary_table_s2.docx]

**Supplementary Table S2**. The information of other gliomas.

| Pathology | Number of patient | Age (range) | Gender (male/female) | IDH-mut |
| --- | --- | --- | --- | --- |
| **Training set** |  |  |  |  |
| Pleomorphic xanthoastrocytoma | 2 | 49-58 | 2 / 0 | No |
| Pediatric⁃type diffuse low⁃grade gliomas | 1 | 56 | 0 / 1 | No |
| Glioneuronal and neuronal tumors | 1 | 44 | 0 / 1 | No |
| Ganglioglioma | 8 | 18-72 | 5 / 3 | No |
| Pilocytic astrocytoma | 2 | 21-32 | 2 / 0 | No |
| Diffuse hemispheric glioma, H3 G34⁃mutant | 1 | 39 | 0 / 1 | No |
| Diffuse midline glioma, H3 K27⁃altered | 2 | 33-50 | 1 / 1 | No |
| Adult⁃type diffuse gliomas (Unclassified) | 3 | 35-49 | 1 / 2 | No |
| **Validation set** |  |  |  |  |
| Pleomorphic xanthoastrocytoma | 1 | 26 | 1 / 0 | No |
| Ganglioglioma | 1 | 46 | 1 / 0 | No |
| Diffuse hemispheric glioma, H3 G34⁃mutant | 2 | 19-31 | 1 / 1 | No |
| Diffuse midline glioma, H3 K27⁃altered | 2 | 37-57 | 2 / 0 | No |
